# Lack of association between classical HLA genes and asymptomatic SARS-CoV-2 infection

**DOI:** 10.1016/j.xhgg.2024.100382

**Published:** 2024-11-02

**Authors:** Eleanor Karp-Tatham, Callum R. O’Neill, Julian C. Knight, Alexander J. Mentzer, Amanda Y. Chong

**Affiliations:** 1 The Centre for Human Genetics, Nuffield Department of Medicine, University of Oxford, Oxford, UK; 2 NIHR Oxford Biomedical Research Centre, Oxford University Hospitals NHS Foundation Trust, Oxford, UK; 3 Chinese Academy of Medical Science (CAMS) Oxford Institute, University of Oxford, Oxford, UK; 4 Ludwig Institute for Cancer Research, Nuffield Department of Medicine, University of Oxford, Oxford, UK

Dear Editor,

We are writing in reference to the recent article published in *HGG Advances* by Marchal et al.^1^ titled “Lack of association between classical HLA genes and asymptomatic SARS-CoV-2 infection,” in which the authors sought to replicate the prior published association between HLA-B∗15:01 and asymptomatic SARS-CoV-2 infection,^2^ but the results were in disagreement, leaving the current field inconclusive. We note that neither Marchal et al.^1^ nor the original study by Augusto and colleagues included data from the UK Biobank (UKB) in their analyses, in which data on asymptomatic SARS-CoV-2 are discernible at scale using the UKB coronavirus serology study (waves 1–6 and wave 7; data categories #994 and #995), the coronavirus infection study (#997), and the self-test antibody study (#998). The UKB cohort offers a well-established, population-based cohort that can add to the current literature on HLA associations with asymptomatic COVID-19 infection. We, therefore, aimed to establish if there are significant associations between HLA-B∗15:01 and asymptomatic infection in the UKB and present the findings here.

Briefly, the UKB is a prospective study cohort of adults living in the UK, aged 40–70+ at recruitment.^3^ The coronavirus serology study ran between July 2020 and February 2022. Approximately 10,000 participants were asked to return six symptom questionnaires along with a finger-prick blood sample over the period of July 2020 to December 2020 (waves 1–6). An additional follow-up questionnaire and sample were collected between November 2021 and February 2022 (wave 7). SARS-CoV-2 seropositivity was determined using anti-S, and later anti-N, laboratory-based serological assays. For a small number of participants, lateral flow and PCR test results were also available through other SARS-CoV-2 serology surveys in the UKB and through linkage with national healthcare databases for England, Scotland, and Wales (Public Health England [PHE], Public Health Scotland [PHS], and Secure Anonymised Information Linkage [SAIL], respectively). All data were accessed under UKB application number 43920.

We defined a SARS-CoV-2 infection as returning a positive COVID-19 test at any time within the 30-day period prior to questionnaire return for waves 1–6 of the questionnaires or in the 12 months leading up to the return of the wave 7 questionnaire. In line with clinical guidelines in the UK, subsequent positive tests within 90 days of the start of any given reporting period were disregarded except for wave 7 due to the longer reporting period. Asymptomatic infections were then defined based on reporting none of the primary COVID-19 symptoms defined in the questionnaires (fever [38°C or greater], wheezing, chills, chest pain, feeling more tired than usual, headache, muscle ache, nausea/vomiting, sore throat, abdominal pain, persistent dry cough, diarrhea, runny nose, loss of sense of smell and taste, shortness of breath, and productive long-term cough [“wet” or chest]) in any returned questionnaires associated with a positive test result. This means that all participants considered asymptomatic had at least one asymptomatic infection, but it does not exclude them from also reporting a symptomatic infection at an earlier or later date. Controls were defined as any individual who reported one or more of the symptoms listed above for all infections in any returned questionnaire associated with a positive test.

HLA types were imputed for UKB participants with genetic data using the multi-ethnic HLA reference panel^4^ (v.1) available through the Michigan Imputation server. HLA alleles were imputed to G-group resolution and later converted to their 4-digit equivalent. Association between HLA alleles (HLA-B∗15:01 and HLA-DRB1∗04:01) and asymptomatic infection was tested using logistic regression in Python 3.9.6. The first 10 genetic principal components were calculated from available genotyping data and included as covariates, along with calculated age at the start of the survey period, reported sex, and immunosuppressed status. Immunosuppressed individuals were classified using International Classification of Diseases 10th Revision (ICD-10) digital codes (Field 41270) relating to chronic viral hepatitis, human immunodeficiency virus (HIV), or human T cell lymphotropic virus (HTLV) infection; organ transplantation; medication; autoimmunity; blood cancer; and congenital disease.

A total of 10,871 participants returned at least one questionnaire over the course of the survey, with 1,624 reporting a positive COVID-19 test. Participants reporting at least one positive test were aged between 50 and 83 at the time of the first questionnaire, with slightly more female (*n* = 820) than male (*n* = 804) respondents. 822 respondents had at least one asymptomatic infection, while the remaining 802 respondents were symptomatic. We also restricted our analyses to the White British subset (WBS) of the UKB, which has been defined based on a combination of genetic ancestry and self-reported ethnicity.^3^ Within the WBS, we identified 508 asymptomatic cases and 520 controls. The frequencies of HLA-B∗15:01 were 0.050 (cases: 0.057, controls: 0.042) in the full cohort and 0.064 (cases: 0.075, controls: 0.052) in the WBS. The frequencies of HLA-DRB1∗04:01 were 0.069 (cases: 0.068, controls: 0.070) in the full cohort and 0.090 (cases: 0.090, controls: 0.090) in the WBS.

We found that individuals carrying HLA-B∗15:01 were significantly more likely to report at least one asymptomatic SARS-CoV-2 infection in both the full cohort (odds ratio [OR; additive model]: 1.480 [1.074–2.038], *p* = 0.016) and the WBS (OR: 1.512 [1.051–2.174], *p* = 0.026). No significant association was found with HLA-DRB1∗04:01 (OR: 1.031 [0.722–1.470], *p* = 0.868) in the full cohort.

We found that the frequency of the HLA-B∗15 allele differed between the cohorts. In the UKB, we found 6% of the whole cohort and 7.5% of the WBS to carry HLA-B∗15 in asymptomatic cases compared to the 17% reported by Augusto et al.^2^ Augusto et al. used self-identified White participants with a 10%–11% higher HLA-B∗15 frequency observed, which could possibly reflect regional differences in allele frequency. Frequency between asymptomatic and symptomatic participants is also larger in their study, with a 10% difference, while in our full cohort, we see an allelic frequency difference of ∼2%, with the respective effect size also being smaller. The frequency in our cohort is in much closer agreement with the Marchal et al. US cohort^1^ (7% asymptomatic and 6% symptomatic). These differences in allele frequencies could also be a direct result of the way that an asymptomatic case has been defined across the studies and their cohorts.

Our asymptomatic definition was in line with the “0 symptoms” strict definition for the US prospective cohort and asymptomatic in the CHGE cohort used by Marchal et al.,^1^ with a greater number of cases (822 vs. 397 cases [86 in the US prospective cohort and 311 in the CHGE cohort]) but less controls. Comparatively, this definition is stricter than the paper presented by Augusto et al.,^2^ in which participants were considered asymptomatic if none of the symptoms persisted for 3 days or longer. UKB questionnaires did not report the duration of symptoms or provide daily symptom reporting, which prevented any direct comparison with these phenotypes in the published papers.

Overall, our analyses, alongside previously published studies,^1^^,^^2^^,^^5^ suggest that the role of HLA alleles in SARS-CoV-2 infection severity is complex. Differences in genetic and non-genetic factors between populations and circulating viral variants may all affect COVID-19 severity.^6^^,^^7^^,^^8^ Our study adds more evidence in favor of HLA-B∗15:01 increasing the likelihood of asymptomatic SARS-CoV-2 infection and encourages further research into the genetic drivers of COVID-19 disease outcomes.

## Acknowledgments

J.C.K. has received support from a 10.13039/100010269Wellcome Trust Investigator Award (grant no. 204969/Z/16/Z) and the 10.13039/501100013373NIHR Oxford Biomedical Research Centre. E.K.-T. is supported by IMMPROVE 10.13039/501100000265MRC grant ref. MY/Y004450/1. The authors also wish to thank the Janssen collaboration funding (R67180/AA001).
